# Edible Fungi Melanin: Recent Advances in Extraction, Characterization, Biological Activity and Applications

**DOI:** 10.3390/jof11100738

**Published:** 2025-10-14

**Authors:** Jiandong Tang, Hebin Shen, Wenyu Lv, Jingxuan Zhang, Junsheng Fu

**Affiliations:** College of Life Sciences, Fujian Agriculture and Forestry University, Fuzhou 350002, China

**Keywords:** edible fungi melanin, eumelanin, extraction, characterization, biological activity, applications

## Abstract

Natural melanin biopolymers exhibit a variety of biological activities, but their commercial development is constrained by numerous factors, including high costs, unsustainable sources, the use of harmful solvents during extraction, and low extraction efficiency. Notably, existing research indicates that synthetic melanin differs from natural melanin in nature, and this difference may directly impact its application efficacy. Additionally, the extraction process itself is highly challenging, primarily due to the diversity and complexity of melanin biopolymer structures. The melanin produced by edible fungi primarily belongs to the eumelanin category. Given its outstanding sustainability and accessibility, it is regarded as an ideal raw material for industrial production. To deepen our understanding of edible fungus-derived melanin and promote its effective application across various fields, a comprehensive review of research on melanin isolated from edible fungi is urgently needed. Such a review will help researchers from different disciplinary backgrounds recognize the importance of edible fungus melanin and provide reference information for their research planning. With this objective in mind, this report reviews the latest research progress in recent years regarding extraction methods, structural characterization, biological activity, and application areas of edible fungus-derived melanin. Additionally, the report explores key characteristic parameters for distinguishing different types of melanin and emphasizes the importance of deepening our understanding of the biosynthetic mechanisms of edible mushroom melanin, aiming to lay the foundation for its efficient production and application in the future.

## 1. Introduction

Edible fungi are a type of large fungus with both medicinal and edible functions. They have been used as folk medicine and health foods since ancient times. Edible fungi are rich in nutrients such as protein, polysaccharides, terpenoids, and lipids, and have various health benefits, including anti-cancer, immune regulation, anti-hypercholesterolemia, anti-viral, anti-diabetic, and anti-inflammatory effects. With the rapid development of the global economy and the growing popularity of green and healthy eating concepts, edible fungi have gained widespread popularity among consumers [1,2,3].

**Figure 1:**
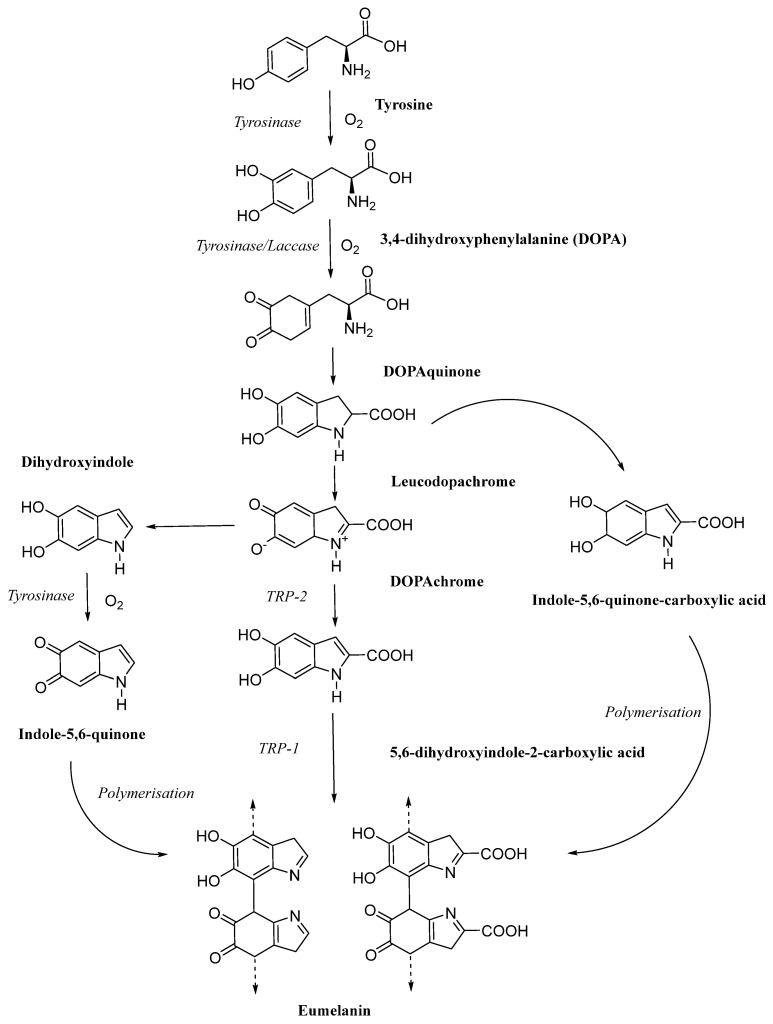
Schematic representation of the eumelanin biosynthesis pathway [7].

Compared with synthetic pigments, natural pigments are favored for their safety and reliability as well as various health benefits. Among them, melanin, as a ubiquitous heterogeneous polymer, exhibits a high degree of diversity in its structure and function [4,5]. Melanin is defined as a heterogeneous polymer produced by the oxidation of phenols and intermediate phenols and the polymerisation of the resulting quinones. Based on its structural monomers, melanin can be divided into three main types: eumelanin, pheomelanin, and allomelanin [6]. The melanin produced by edible fungi is predominantly classified as eumelanin. Eumelanin is a melanin that ranges in color from black to brown. Its biosynthesis begins with the catalysis of tyrosine by tyrosinase and is completed via the L-dopa pathway (Figure 1) [7]. Melanin, named by Swiss scientist Berzelius [8], is a class of macromolecular substances formed by the polymerisation of polyhydroxyindoles or polyhydroxyphenols [9]. Its structure is complex and diverse, making it insoluble in water, most organic solvents, and acidic solutions, but soluble in alkaline solutions. Additionally, melanin readily forms strong bonds with macromolecules such as proteins, carbohydrates, and tannins, and exhibits amorphous properties. These characteristics thus make it difficult to accurately determine their precise chemical structure and composition, resulting in a still limited comprehensive structural characterization to date [10]. Natural melanin is a unique and rare type of natural pigment, exhibiting excellent photothermal stability and being the only known endogenous radiation-protective agent with radiation-protective activity. This pigment is widely distributed in nature and is the most common and abundant pigment in the biological world. With advances in melanin research, its numerous biological activities have been discovered, including antioxidant, free radical scavenging, anti-radiation, anti-ageing, and immune-boosting properties. These properties have led to its widespread application in fields such as food, cosmetics, medicine, and new materials [11,12]. Currently, research into the application of synthetic melanin analogues such as polydopamine is advancing steadily, with its potential garnering significant attention within the biomaterials field. However, from the perspective of green and sustainable development, melanin derived from edible fungi offers an alternative pathway with greater ecological value. This natural melanin not only facilitates large-scale acquisition through biological cultivation but also possesses biodegradability unmatched by synthetic materials. This establishes its foundation for applications in fields demanding high biosafety, serving as a potent natural supplement and alternative to polydopamine [13,14]. At present, research on edible mushroom melanin mainly focuses on its extraction and purification techniques, physicochemical properties and biological activity evaluation [15,16,17,18,19,20,21,22]. There are bottlenecks in developing environmentally friendly and economically efficient eukaryotic source melanin production technology. In contrast, strategies based on microbial fermentation are showing great application potential [23,24]. This process is simple, and by optimising key process parameters that influence melanin synthesis, production can be significantly increased. Clarifying the relative competitive advantage of edible fungi-derived melanin within the natural pigment system is a critical prerequisite for its application assessment. Compared to animal-derived sources, its primary advantage lies in sustainability: edible fungi cultivated through artificial methods do not rely on biological resource harvesting, are not constrained by season or geography, and avoid animal ethics controversies. Compared to other microbial sources, their fundamental assurance lies in safety. Most edible fungi belong to safe strains, making their melanin more readily compliant with stringent regulatory requirements in food and pharmaceutical sectors while encountering fewer public perception barriers. Although their production efficiency may fall short of certain optimized bacterial strains, their exceptional sustainability, reliable safety profile, and favorable social acceptance collectively establish edible fungi-derived melanin as a solid, ideal natural alternative in high-value applications—particularly within the food and pharmaceutical industries. Currently, there have been some outstanding reviews published on melanin, focusing on specific attributes such as biosynthesis, extraction, characterisation, production prospects, and its biomaterial applications [5,25,26,27,28,29,30]. However, there is still a lack of comprehensive reviews on melanin derived from edible fungi.

This review focuses on melanin derived from edible fungi and is one of the first reviews in this field. It emphasises the extraction, characterisation, biological activity, and application of melanin. This review fills the gap in systematic research on melanin derived from edible fungi and provides important theoretical references for its in-depth development and utilisation.

## 2. Extraction and Purification

The vast majority of natural melanins are poorly water-soluble, but they are soluble in strong alkaline solutions (pH ≈ 11) and completely precipitate in acidic solutions (pH < 3) [31]. This solubility characteristic is closely related to the interaction between the surface charges of melanin monomers and the solution. Specifically, in neutral to alkaline environments, the negative surface charges of monomers undergo further deprotonation, leading to enhanced electrostatic repulsion between monomers, thereby improving the dispersion and solubility of the polymer. Conversely, under low pH conditions, protonation weakens the surface electrostatic charges, promoting monomer polymerisation and triggering precipitation [32]. This property provides a theoretical basis for the extraction of edible fungus melanin. In addition to the alkali-soluble acid precipitation method, organic solvent extraction and enzymatic hydrolysis are also commonly used extraction methods and can be applied synergistically to improve extraction efficiency.

**Figure 2:**
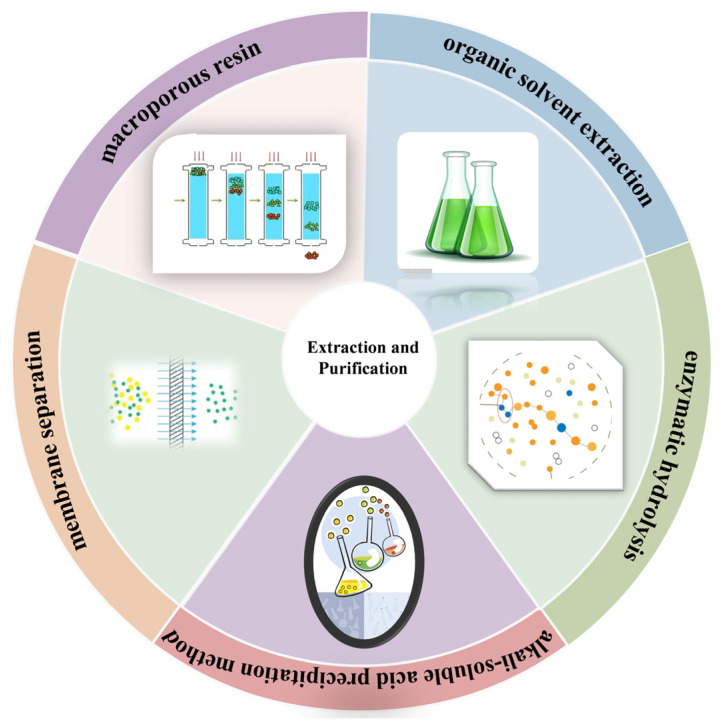
The method of extraction and purification.

Currently, research on edible mushroom melanins primarily focuses on crude extracts. Additionally, their industrial applications remain limited, as the natural melanins extracted are often unstable and prone to degradation due to their structural characteristics and environmental factors. Therefore, conducting in-depth characterisation of the structure of edible mushroom melanins and investigating their stability is crucial for fully exploiting edible mushroom resources and providing theoretical support for natural pigment research. Clarifying the composition and structural characteristics of edible mushroom melanins and reducing the difficulty of structural characterisation are essential. In this process, the use of membrane filtration and macroporous adsorption resin technology for the separation and purification of melanin crude extracts is of critical importance. The method for extraction and purification can be seen in Figure 2.

### 2.1. Solvent Extraction Method

The solvent method uses inorganic or organic solvents that are insoluble in melanin (such as deionised water, quaternary ammonium compounds) as extraction media. Organic solvent extraction commonly employs strong hydrogen bond solvents, which can compete with hydrogen bonds in biological polymers such as cellulose, keratin, and agarose in the crude extract, disrupting the protective layer formed around the melanin and dissolving it, thereby releasing the melanin. Additionally, under alkaline conditions, organic solvents can form hydrogen bonds with the hydroxyl, carboxyl, and imino groups of melanin, promoting the separation of melanin monomers and facilitating the removal of impurities [33,34]. Soak the raw material in a large amount of deionised water or organic solvent and mix thoroughly to dissolve soluble substances such as ionic salts, proteins, and polysaccharides; then centrifuge to collect the melanin precipitate; finally, freeze-dry the precipitate to obtain a melanin extract free of soluble impurities [33,35]. This method maximises the retention of the natural structure and properties of melanin. The extraction waste, rich in proteins and sugars, can be directly used as fertiliser. However, the processing conditions are mild, the cost is low, and the process is time-consuming, making it difficult to break the covalent cross-linked structures in the crude extract, resulting in incomplete impurity removal and reduced extraction efficiency [36].

### 2.2. Alkali Extraction and Acid Precipitation Method

Alkali extraction and acid precipitation are the most commonly used methods for extracting and purifying melanin [37,38,39]. Solubility is the most conventional step for detecting and characterizing melanin. Due to its unique solubility, melanin is usually insoluble in water and most organic solvents, but soluble in dimethyl sulfoxide (DMSO) and alkaline aqueous solutions, and precipitates under acidic conditions. This property makes it suitable for the extraction process of melanin [5]. The crude extract was obtained from the fermentation broth of *Phlebopus portentosus* using the alkali solubilisation and acid precipitation method, with an extraction rate of 0.44 g/L [17]. The melanin of *Ganoderma lucidum* was extracted and purified using the alkali solubilisation and acid precipitation method. The differences in the structure and physicochemical properties of the melanin in *G*. *lucidum* fruiting bodies, mycelium and spores were investigated [40]. This method, combined with ultrasound-assisted extraction, has been widely used in the extraction of melanin from various edible fungi [22,41,42,43,44,45,46,47]. Although this method can effectively remove impurities, overly harsh reaction conditions, especially prolonged exposure to strong acids and bases, may cause melanin to undergo decarboxylation, destroying its original chemical structure. This not only partially loses the inherent functions of natural melanin but may also introduce new impurities and reduce the extraction rate [26,48]. In addition, the waste liquid produced during the extraction process causes environmental pollution, and its subsequent treatment remains to be resolved.

### 2.3. Enzymatic Hydrolysis Method

Enzymatic hydrolysis can effectively promote hydrolysis and release intracellular pigments and other components by breaking down the cell wall. Since its first application in 2004, it has demonstrated multiple advantages [5,49]. It is an extraction method that combines protective properties with efficient impurity removal. This method utilizes biological enzymes (such as alkaline proteases) to break down biological tissue cells and release melanin, or to hydrolyse peptides on melanin particles for purification. Compared to solvent-based methods, enzymatic hydrolysis achieves superior impurity removal; compared to alkaline solubilisation and acid precipitation, its reaction conditions are milder, maximizing the retention of melanin’s natural molecular structure. The extraction of edible mushroom melanin involves the use of specific enzymes and enzyme complexes to break down cell walls, mainly cellulase, pectinase, and papain, the efficiency is satisfactory [45,50]. Enzymatic hydrolysis of edible mushroom melanin holds great potential and is regarded as a safe and environmentally friendly alternative, especially due to its efficient and mild extraction characteristics, although further research is still needed in this area.

### 2.4. Other Auxiliary Methods

Cavitation extraction is widely used in pigment extraction due to its environmental friendliness. This technology utilises the cavitation phenomenon, where rapid changes in pressure within a liquid medium create small low-pressure vapour-filled cavities within cells. The collapse of cavitation bubbles releases energy, enhancing mass transfer across interfaces and enabling solvents to penetrate cellular materials more effectively. A variety of cavitation techniques have become effective means to achieve efficient extraction, such as ultrasonic-assisted extraction (UAE), negative pressure cavitation (NPC) extraction, microwave-assisted extraction (MAE), and hydrodynamic cavitation extraction (HCE) [5,51,52]. Solid-state fermentation was used to produce melanin from *I*. *hispidus*, and an ultrasonic-assisted extraction process was optimised to improve the extraction efficiency. Compared with the non-ultrasonic control group, the extraction rate increased by 37.33% [22]. Ultrasound-assisted alkaline cellulase treatment was also used to process *Auricularia auricula*, and based on single-factor experiments, the Box–Behnken design was used to optimise the optimal cell wall breaking process parameters. After optimisation, the yield of melanin from *A. auricula* reached 3.201% ± 0.018% [53]. Previous studies have reported that edible mushroom waste (such as *A. auricula* residue) can be used to recover melanin through an UAE process. This melanin dissolves well in alkaline solutions and exhibits certain thermal stability [19].

Traditional media optimisation methods optimise only a single factor at a time, which is a cumbersome and time-consuming process. Additionally, these methods often overlook the interactions between factors, leading to inconsistent results. Emerging statistical experimental design tools overcome these limitations. The Plackett-Burman design (PBD) can simultaneously screen multiple factors to identify key factors, eliminate non-significant factors with minimal influence on the response variable, and retain significant factors for further optimisation. Based on the significant factors identified by PBD, Centre Composite Design (CCD) and Response Surface Methodology (RSM) can be used to establish models describing the relationships between variables and responses [54,55,56]. Given the widespread application and high demand for melanin, there is an urgent need to develop low-cost, high-yield production processes using such statistical methods. By employing PDBs and CCD to statistically analyse angles and using response surface methodology for analysis, this study investigated the enhancement of melanin production in Auricularia auricula. Under optimised conditions, melanin production reached 1008.08 mg/L, representing a 3.29-fold increase [57]. Based on single-factor experiments and response surface analysis, the optimal conditions for extracting melanin from the fruiting bodies of *Auricularia auricula-judae* (Hei 29) were determined. Under the optimized conditions, the melanin yield reached 2.59% [58]. This auxiliary extraction study was also used in the solid-state fermentation production of *I*. *hispidus* melanin [22].

### 2.5. Efficient Preparation Exploration Strategy

Melanin extraction is highly challenging due to the structural diversity and complexity of its biopolymer nature. Traditional methods are inefficient, resulting in low yields and high costs, which limit the widespread application of melanin. Therefore, there is an urgent need to develop sustainable methods for the efficient and selective extraction of melanin from natural sources [25,59]. Quaternary ammonium salt-based ionic liquids and deep eutectic solvents (DESs) are emerging as highly effective media for dissolving biopolymers such as cellulose, carrageenan, agarose, and pigments [33,60,61,62,63]. Quaternary ammonium salts act as hydrogen bond acceptors (HBAs) and can form hydrogen bond networks with hydrogen bond donors (HBDs) such as alcohols, acids, and sugars. Given that quaternary ammonium salt solutions contain carboxyl and hydroxyl groups, it is anticipated that they can form favourable hydrogen bonds with melanin, thereby enabling its efficient extraction in the solvent. Biological synthesis is also an important pathway for melanin production. Studying its mechanisms not only has profound theoretical significance but also broad practical application value. In this context, melanin derived from fungi, with its significant advantages in sustainability and accessibility, has become a highly promising raw material option for industrial production [64].

**Figure 3:**
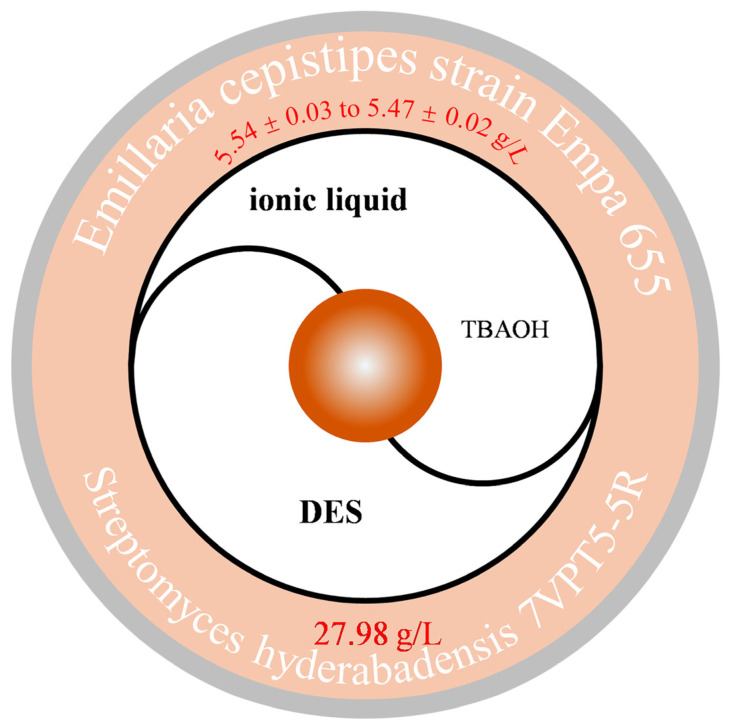
New explorations of edible fungi in ionic liquids and low melting point solvents.

Previous studies have investigated the suitability of a water solution of tetrabutylammonium hydroxide (40% *w*/*w* TBAOH in water) for extracting melanin biopolymers from *Streptomyces hyderabadensis* 7VPT5-5R. Compared to traditional extraction methods, the TBAOH water solution extraction method increased melanin yield by 66%. The used solvent was recycled five times, resulting in melanin yields ranging from 5.54 ± 0.03 to 5.47 ± 0.02 g/L. Considering the efficiency of hydrated TBAOH in effectively extracting melanin and its potential for recycling, it can be considered a sustainable solvent for large-scale extraction of pigments from microbial cultures [33] (Figure 3). This method has also been reported in the field of edible fungi. The cell walls of *Auricularia auricula* fruiting bodies are extremely tough, and traditional preparation methods struggle to dissolve melanin. Therefore, ultrasonic-assisted alkaline cellulase was first employed, and optimal cell wall disruption parameters were optimised using a Box–Behnken design based on single-factor experiments. After optimisation, the melanin yield from *A*. *auricula* reached 3.201 ± 0.018%. Subsequently, further extraction was conducted using different types and ratios of DES. When choline chloride and urea were selected in a 1:2 ratio, the melanin yield reached as high as 25.99% ± 2.36% [53] (Figure 3). In studies on melanin biosynthesis using *L*-tyrosine as a precursor, we evaluated the production capacity of different fungi. The results showed that the *Armillaria cepistipes* strain (Empa 655) had the highest melanin yield, reaching 27.98 g/L. Physical and chemical characterization of the obtained melanin confirmed its typical eumelanin structure. The biosynthetic method proposed in this study is efficient, scalable, and sustainable, demonstrating significant application potential and providing support for future technological development [46].

The integration of multiple extraction techniques can produce synergistic effects, enhance extraction efficiency, and lay the foundation for the development of more advanced and efficient technologies. Each extraction method has its specific advantages and limitations. Given the diverse sources and amorphous nature of melanin, there are currently no standardised extraction and purification methods. Therefore, through the systematic integration and targeted optimisation of extraction methods, production media, and fermentation conditions, it is possible to effectively regulate the yield and type of the final product.

## 3. Characterization of Melanin

**Figure 4:**
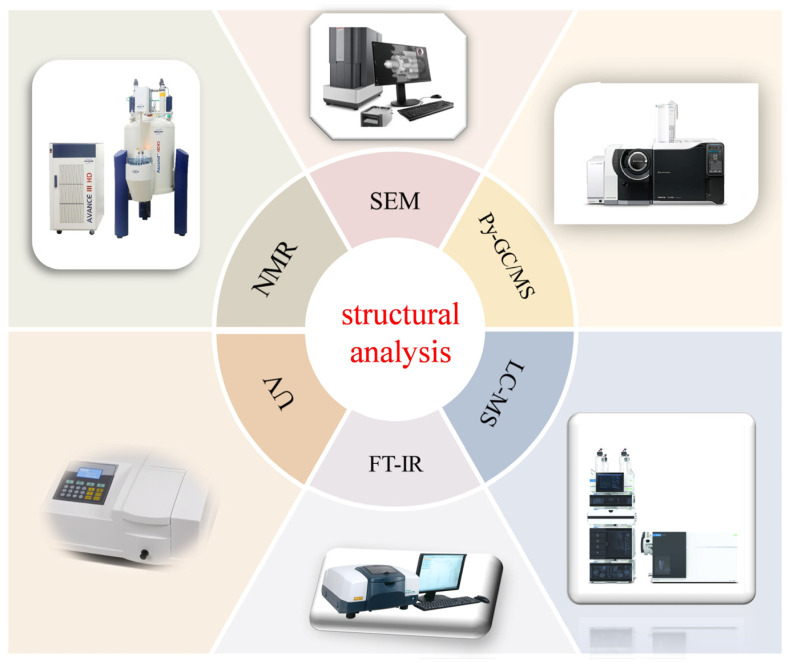
Different characterization methods for identification and structure elucidation of melanin.

Due to the heterogeneous nature of melanin, it lacks a unique and well-defined structure. Therefore, a series of rigorous characterisation techniques (Figure 4) are required to patiently determine the structure of melanin and distinguish between its two main types [65].

### 3.1. UV–Visible Spectroscopy

UV radiation triggers the formation of pyrimidine adducts, thereby inhibiting transcription and translation processes. Organisms can effectively defend against these UV rays through the radiation absorption mechanism of melanin [66]. Natural melanins are rich in conjugated structures, chromophores, and auxiliary chromophores. Their ultraviolet absorption characteristics are commonly used for preliminary identification and characterisation. In the ultraviolet region, natural melanins exhibit strong absorption characteristics, with typical absorption spectra showing that the absorption value decreases with increasing wavelength from the maximum absorption peak. Furthermore, plotting the logarithm of the absorbance against wavelength yields a straight line with a negative slope. Negative slope as an indicator for qualitative detection of melanin [67,68,69,70]. The *A*_650_/*A*_500_ ratio can be used to quantitatively assess the relative content of eumelanin in total melanin. Generally, a ratio higher than 0.25 indicates that the sample is rich in eumelanin; while a ratio lower than 0.15 suggests a higher content of brown melanin [71].

The medicinal fungus *Ophiocordyceps sinensis* exhibits maximum absorbance at 237 nm and a typical melanin structure [72]. The melanin in *Tremella fuciformis* bran exhibits a maximum characteristic absorption peak at 318 nm. The logarithmic value of the melanin absorbance forms a linear curve with a slope of −0.0032 with respect to wavelength, indicating that the melanin in *T*. *fuciformis* bran possesses the typical characteristics of melanin [16]. Similarly, the absorption amount of melanin in *I. hispidus* decreased almost linearly with the increase in wavelength. The relationship between absorbance and the logarithm of wavelength presented a straight-line curve with a negative slope of −0.0031, which is a typical characteristic of melanin. No absorption peaks were found at 260 cm and 280 cm, indicating that the contents of nucleic acid and protein impurities were extremely low [22]. The ability of melanin to absorb ultraviolet rays stems from its complex conjugated molecular structure. Moreover, eumelanin and brown melanin can be distinguished based on their spectral characteristics under UV irradiation. This method has been widely used in the characterization of edible fungi melanin [73,74,75].

### 3.2. FT-IR

Compared with UV spectroscopy, FT-IR can provide information on the functional groups and categories of compounds. After Bonner and Duncan first applied infrared spectroscopy to characterize the structure of melanin, this method has been widely adopted and has now become the best means to analyze the functional groups and chemical bonds of melanin. The significant characteristic absorption peaks of natural melanin at 1600–1700 cm^−1^ and 3300–3500 cm^−1^ indicate the presence of carbonyl groups and amino or hydroxyl groups [46,76]. The structure of eumelanin composed of polymeric units of indole-5,6-quinone and 5,6-dihydroxy-indol-2-car boxylic acid, and pheomelanin comprising benzothiazines and ben zothiazoles units in the structure [6]. The black pigment of *Auricularia heimuer* is located at 1652.71 cm^−1^ and is attributed to C=O stretching or aromatic C=C stretching; the N-H bending vibration at 1540.00 cm^−1^ and the C-N stretching vibration at 1402.39 cm^−1^ indicate that the black pigment has a typical indole structure; the weak absorption band at 627.45 cm^−1^ suggests that the aromatic ring has undergone substitution and formed a conjugated system [50]. And based on the similarity of infrared spectra, the functional groups suggest that the melanin in the *Ganoderma lucidum* fruiting body, mycelium and spores might be eumelanin [40]. In several studies on edible fungi, the melanin was identified as eumelanin through FT-IR analysis [16,73,77]. These results are in line with the usual characteristics of melanin.

### 3.3. Physicochemical Properties

The most salient characteristics of natural melanin are its solubility, stability, light absorption properties, redox properties, metal chelation and ion exchange properties.

Melanin has unique solubility properties: it is generally insoluble in water and many organic/inorganic solvents, but soluble in DMSO and alkaline aqueous solutions, and precipitates under acidic conditions. This characteristic renders it an ideal method for extracting melanin. In terms of its stability, it is widely accepted that the highly conjugated structure of natural melanin confers upon it light and thermal stability, and that it is relatively stable in alkaline media. The stability of the system is influenced by the presence of certain metal ions. However, melanin has been observed to form a precipitate in acidic solutions and can be oxidised and faded by strong oxidants, such as hydrogen peroxide, potassium permanganate, potassium dichromate and sodium hypochlorite. Melanin’s most notable property is its capacity for strong light absorption, which is ascribed to its high conjugation effect. Melanin has been demonstrated to exhibit strong light absorption capabilities across a wide spectral range (150–800 nm). As the wavelength increases from the ultraviolet region to the visible light region, the light absorption values gradually decrease, and the maximum absorption wavelength is approximately 210 nm. As previously stated, the infrared spectral region of the spectrum is also characterised by characteristic absorption peaks in natural melanin. Melanin exhibits significant redox capabilities, which mainly stem from the abundant semiquinone radicals free radicals present in its molecular structure. These free radicals remain stable within a wide pH range, enabling melanin to act both as an electron donor and an electron acceptor, participating in various monovalent or divalent redox reactions. Moreover, the catechol (o-cresol) and phenolic units in the melanin molecule have strong affinity for metal ions, endowing it with excellent metal ion binding ability. Studies have shown that metal ions can form coordination bonds with the carbonyl, hydroxyl, and amino functional groups of melanin, thereby firmly binding to it [5,25,27].

The chemical analysis method usually includes elemental analysis, functional group determination and chemical degradation. Elemental analysis is used to study the elemental composition of natural melanin, mainly including C, H, N, S and O. Different types of melanin have differences in elemental content. Eumelanin contains N but no S, phaeomelanin contains N and S, allomelanin melanin contains neither N nor S. However, since natural melanin is usually a mixture of two or more types of melanin, elemental analysis can only make a preliminary judgment on its types. In addition, the H/C, O/C and N/C atomic ratios of melanin in the elemental analysis are also often used to determine its type and purity [23]. Studies have shown that the melanin in the fruiting bodies, mycelium, and spores of *Ganoderma lucidum* are composed of C, H, N, O, and S, with S content of 0.50%, 0.51%, and 0.63%, respectively. This indicates that the primary component of the melanin in the fruiting bodies, mycelium, and spores of *G*. *lucidum* is eumelanin [40]. The melanin in *B*. *griseus* can be classified as eumelanin and contains trace amounts of pheomelanin [47].

Structural analysis based on chemical degradation methods is helpful for eumelanin and pheomelanin. In this method, pigments are treated with strong oxidizing agents or strong reducing agents, and the degradation products are separated and purified by chromatography and analysed by spectrophotometry [78]. Chemical degradation methods not only play an important role in analyzing the structure of melanin but also have important practical value in identifying the types of melanin in tissue samples through the identification of characteristic degradation products.

### 3.4. Pyrolysis-Gas Chromatography/Mass Spectrometry (Py-GC/MS)

Py-GC/MS is an effective method for analyzing melanin. The characteristic degradation products of eumelanin are primarily pyrrole, indole, and their alkyl derivatives, which serve as indicators of its biosynthetic precursor units [78]. Eumelanin synthesised from catecholamine precursors also releases intact catechol (1,2-dihydroxybenzene). In contrast, the characteristic degradation products of pheomelanin are heterocyclic compounds containing cysteine, as well as specific isomeric amino hydroxyphenylalanine and amino hydroxyphenyl ethylamine pigment markers [79,80]. Py-GC/MS technology has been used to analyse the structure of melanin in several edible fungi. The results indicate that the melanin in edible fungi is primarily true melanin, providing scientific basis for the precise analysis of the structure of melanin in edible fungi [47,81,82].

### 3.5. Nuclear Magnetic Resonance (NMR)

NMR technology is based on the quantum magnetic properties of atoms at the atomic scale. By analysing chemical shifts and peak splitting patterns, it can determine the bonding state of hydrogen and carbon atoms in a substance. Using nuclear magnetic resonance spectroscopy, the bonding conditions of carbon and nitrogen atoms in the structure of melanin can be accurately and efficiently determined.

In the aliphatic region of the ^1^H NMR spectrum, the signal peaks in the 0.0–2.5 ppm range can be assigned to C–H stretching vibration signals from alkyl fragments, which may originate from residual proteins [23]. Additionally, at 3.7–4.2 ppm, peaks associated with –CH_2_ or –CH_3_ groups linked to N or O atoms are observed [83,84]. In agreement with the aliphatic region, the signals observed in the aromatic region between 7.2 and 8.0 ppm correspond to the indole ring within the melanin framework, reflecting the chemical environment of aromatic hydrogen atoms and the varied bonding patterns among different functional groups in melanin. The signal peaks in the 4.2–5.4 ppm range are caused by C=C–H groups connected to N atoms and/or oxygen atoms. The signals in the 5.5–6.5 ppm range indicate the presence of indole –NH groups. The peak signals at 8.0–8.5 ppm indicate the presence of pyrrole–CH groups of indoles substituted with carboxyl groups [10,21,47,85]. In the ^13^C NMR spectrum, the signal peaks of aliphatic C-H carbon atoms appear in the range of 35–50 ppm; aromatic carbon atoms appear in the 110–160 ppm range; and signals in the 165–175 ppm range may be attributed to the carbonyl groups of the main chain and side chains of peptide bonds, as well as the carbonyl groups of the melanin quinone portion [21,47,86]. Due to the heterogeneity and amorphous nature of some edible mushroom melanins, as well as their insolubility in aqueous buffers, specialised techniques such as solid-state NMR are required to elucidate the composition of melanins [18,87].

### 3.6. Electron Paramagnetic Resonance (EPR)

EPR spectroscopy primarily studies chemical free radicals, transition metal ions, and their compounds. By observing their spectra, information about unpaired electron states and their environment can be obtained, making it an important tool for exploring the microscopic structure and motion states of substances. This technique has revealed the presence of stable quinone free radicals in melanin. Therefore, detecting whether a substance contains such stable free radicals can serve as a basis for identifying melanin using EPR spectroscopy. The paramagnetic properties of melanin molecules originate from their quinone groups [88,89].

Eumelanin generates ortho-quinone free radicals, whose EPR spectra exhibit a single peak with hyperfine structure even at high power; the EPR spectra of pheomelanin, however, are structurally complex and lack distinct hyperfine features due to the interaction between free radical electrons and nearby nitrogen atoms. Natural melanins can be distinguished from synthetic melanins through their characteristic EPR spectra: their spectra exhibit mildly asymmetric singlet lines (g ≈ 2.003–2.004), lack hyperfine structure, and have peak line widths of approximately 7 G, indicating the presence of stable carbon-centered free radicals [46]. The EPR spectrum of melanin from *O*. *sinensis* shows a strong, slightly asymmetric single line, indicating the presence of stable organic free radicals, g = 2.00508 [72]. Additionally, the spectrum of melanin in *I*. *hispidus* is slightly asymmetrical with a single peak and no superfine structure, with g = 2.005 [22]. The EPR characterisation results of melanin in *Pleurotus cystidiosus* and *Auricularia auricula* were also validated [90,91].

### 3.7. Surface Morphology

The morphology and particle size distribution of melanin are typically characterised using microscopy techniques such as scanning electron microscopy (SEM), transmission electron microscopy (TEM) or atomic force microscopy (AFM).

**Figure 5:**
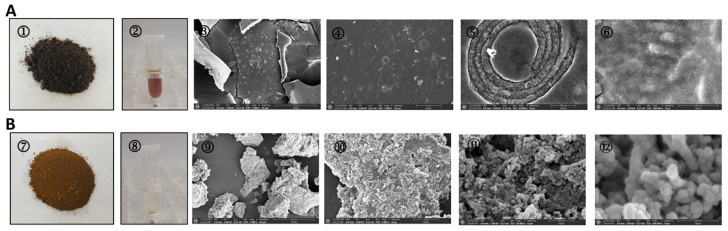
Morphological characteristics and scanning electron micrographs of the melanin obtained from I. hispidus. (**A**): Water-soluble IHFM: ① water-soluble melanin powder; ② 5 mg of powder dissolved in 1 mL of water; ③–⑥ microstructure of melanin, which is surrounded by concentric layers of circular units. (**B**): Water-insoluble IHFM: ⑦ water-insoluble melanin powder; ⑧ The latter is insoluble in water, and there is a powder residue at the bottom of the centrifuge tube; ⑨–⑫ microstructure of melanin, with an irregular arrangement [21].

**Figure 6:**
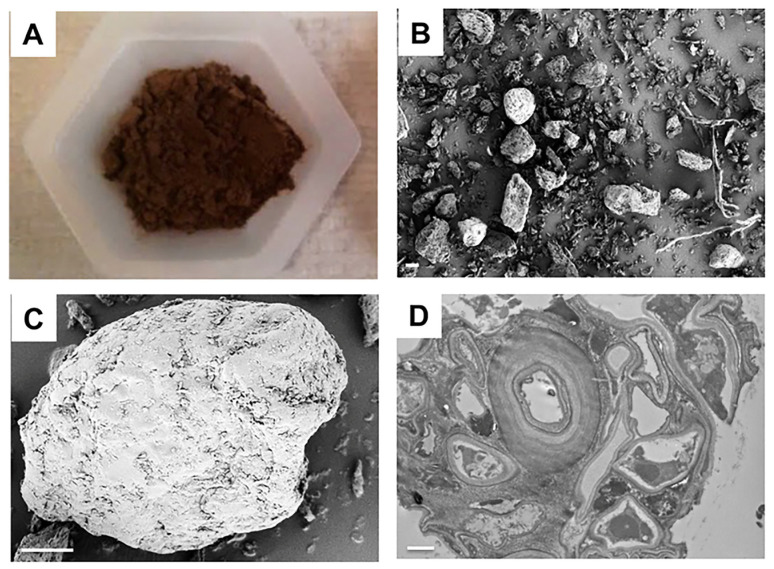
Electron microscopy analysis of the *A. auricula* mushroom powders (**A**). *A. auricula* mushroom preparations were processed for scanning electron microscopy (SEM, (**B**,**C**)) and transmission electron microscopy (TEM, (**D**)). Scale bars in (**B**,**D**) are 10 μm, and scale bar in (**C**) is 1 μm [20].

By observing and analysing the ultrastructure of melanin in *I. hispidus*. The results indicate that the microscopic structures of water-soluble and insoluble *I. hispidus* melanin exhibit significant differences. Water-soluble *I. hispidus* melanin has a smooth surface composed of spherical particles with diameters of 10–15 nm, which are arranged in irregular planes; insoluble melanin contains circular units enclosed within heterogeneous aggregates surrounded by thick-walled concentric layers [21] (Figure 5). In addition, SEM images of melanin from *Auricularia auricula* show a non-uniform structure with amorphous clumps and no crystal structures observed [53]. In addition, electron microscopy studies show that *A. auricula* melanin is associated with the mushroom cell wall in a manner similar to that of melanin from the model fungus *C. neoformans* [20] (Figure 6).

### 3.8. Liquid Chromatograph Mass Spectromete (LC-MS)

Since melanin is insoluble in water and most organic solvents, its structural analysis typically relies on chemical degradation methods. LC-MS technology can infer the molecular weight and possible molecular structure of unknown compounds by analyzing ion fragment information. Therefore, LC-MS analysis can further confirm the structure of melanin.

**Figure 7:**
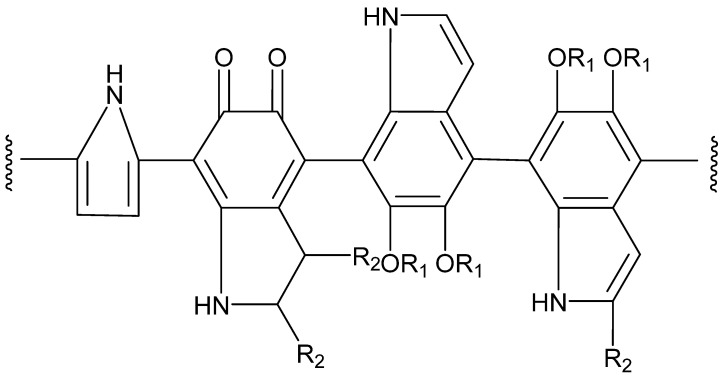
The structure of BgM. R_1_ = H/alkanes/alcohols/fatty acid; R_2_ = CH_3_/COOH [47].

Domestic scholars used UPLC-MS/MS analysis to infer the structure of *Boletus griseus* melanin (BgM) and identified four main primary mass peaks, inferring their molecular formulas to be C_8_H_7_O_2_N, C_9_H_7_O_4_N, C_13_H_8_O_4_N_2_, and C_14_H_8_O_6_N_2_. Based on this, the possible chemical structure can be inferred as shown in Figure 7. This structure is mainly composed of 5,6-dihydroxyindole and its derivatives, which are combined with small molecules such as alkanes, alcohols, and fatty acids. These components are polymerized through various chemical bonds to form macromolecules [47]. In addition, Meanwhile the condensed molecular formula ([C_18_(OR)_3_H_7_O_4_N_2_]*_n_*) and structural formula (Figure 7) of *A. auricula* melanin were concluded based on UV–Vis, HPLC, FT-IR, NMR and elemental assay. This is a eumelanin and also a macromolecular polymer of 5,6-dihydroxyindole and 5,6-dihydroxyindole-2-carboxylic acid [23].

Compared to its widespread application in the study of plant and animal-derived melanins, the UPLC-Q-TOF-MS/MS technique has seen relatively limited systematic research reports in the field of edible mushroom melanin fine structure analysis. UPLC-Q-TOF-MS/MS, with its ultra-high resolution, precise mass determination, and robust multi-level fragmentation analysis capabilities, offers unique advantages in revealing the monomer composition, linkage patterns, and polymerization characteristics of complex melanin molecules. However, systematic reports on the application of this technology to investigate the chemical essence of melanin in edible fungi, an important group, remain scarce. Given the potential bioactive value and structural diversity of edible mushroom melanins, focusing on the application of UPLC-Q-TOF-MS/MS technology to deeply analyze their structural characteristics and structure-activity relationships is undoubtedly a critical research direction and important breakthrough area that urgently needs to be strengthened in this field.

Multiple analytical techniques provide key tools for accurately characterising edible mushroom melanins. Solubility and ultraviolet spectroscopy enable rapid preliminary screening and identification of melanins. Specific techniques can effectively distinguish between different types of melanins. Therefore, to fully characterise structurally heterogeneous melanins, it is necessary to comprehensively apply the aforementioned techniques.

## 4. Biological Activity

**Figure 8:**
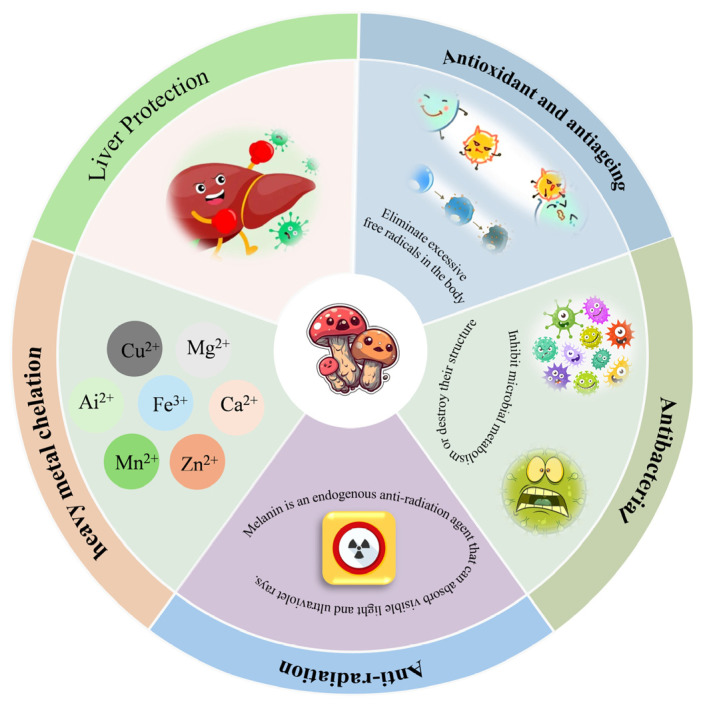
The biological activity of melanin derived from edible fungi.

Research has found that melanin possesses multiple biological activities, such as antioxidant, antiageing, antibacterial, anti-radiation, chelation of heavy metals, and liver protection (Figure 8).

### 4.1. Antioxidant and Antiageing

Free radicals contain unpaired electrons and are extremely active, with strong oxidising properties. Excessive free radicals can oxidise and attack biological macromolecules such as cell membranes, nucleic acids, proteins, and enzymes, leading to changes in membrane properties, gene mutations, and reduced enzyme activity, which in turn can cause cell dysfunction, ageing, and even death. Therefore, it is important to study natural antioxidants that can eliminate free radicals.

Research has shown that mushroom melanin possesses excellent antioxidant and anti-ageing properties. Among these, the melanin from *Gomphidius viscidus* exhibits a typical melanin molecular structure and demonstrates excellent antioxidant capacity both in vitro and in vivo, as well as anti-ageing and stress-resistant properties [92]. Meanwhile, the melanin from the fruiting bodies of *A*. *aegerita* also possesses certain reducing capacity, DPPH radical scavenging ability, and Fe^2+^ chelation capacity [43]. Similarly, melanin from the fruiting bodies of *Auriculair auricuia* exhibits excellent free radical scavenging capacity, with IC_50_ values of 0.48 mg/mL and 0.176 mg/mL against hydroxyl radicals (OH) and DPPH radicals, respectively [93]. Additionally, components F1 and F2 in the melanin of *Auricularia auricula* exhibit a scavenging efficiency of over 80% for superoxide anion radicals (O_2_^−^·) and approximately 40% for hydroxyl radicals [94,95]. There was no significant difference in the ABTS, DPPH, and ·OH scavenging free radical between the control group melanin and the waste residue melanin of *A. auricula*. Notably, waste residue melanin significantly inhibited H_2_O_2_-induced cell death. At a concentration of 1.6 mg/mL, cell viability recovered to 98.09 ± 5.97%. Further morphological observations confirmed that this melanin effectively alleviated cell morphological damage caused by oxidative stress [19]. In terms of extraction, melanin extracted from *Auricularia heimuer* using a cellulase-ultrasonic co-extraction method demonstrated stronger scavenging activity against ABTS, DPPH, and hydroxyl radicals compared to melanin extracted without cellulase [96].

### 4.2. Antibacterial

Bacterial infections are one of the major challenges to human health, and bacterial resistance to traditional antibiotics has become a global concern. Meanwhile, with its good biocompatibility and easy functionalization, melanin has been proven to be an effective antibacterial agent, especially for treating local infections [97].

Research has found that melanin extracted from *T*. *fuciformis* bran (3.2 mg/mL) exhibits an inhibition rate exceeding 90% against most test bacteria, with particularly effective inhibition against *S*. *aureus* compared to other Gram-negative bacteria [15]. In addition, bioactive melanin components isolated from Chaga were studied, and the results showed that the isolated melanin components have good antibacterial activity [98]. Additionally, in terms of melanin modification. The study investigated the modification of melanin extracted from *Auricularia auricula* using arginine. The minimum inhibitory concentrations (MIC) of melanin and Arg-melanin against *S*. *aureus* were 2.5 mg/mL and 1.5 mg/mL. Arg-melanin exhibited enhanced water solubility and antibacterial activity, providing a theoretical basis for the application of melanin in the food and pharmaceutical industries [99].

### 4.3. Anti-Radiation

Melanin is an endogenous anti-radiation agent that absorbs visible light and ultraviolet light. It is currently the only known natural substance that protects the skin from radiation damage and reduces radiation damage to cells in the body [100]. The radiation resistance of melanin is mainly manifested in two mechanisms: one is to block external radiation through absorption or scattering; the other is to eliminate light-induced free radicals by utilising its free radical scavenging properties, thereby protecting the body.

Research has shown that in mice exposed to high doses of radiation, supplementation with *Auricularia auricula-judae* melanin protected 80% of individuals from lethal doses of radiation, while the untreated group died from gastrointestinal syndrome, confirming the significant radiation-protective effects of this melanin [101]. Additionally, *O*. *sinensis* melanin also demonstrated strong UV-blocking capabilities [72]. Currently, research on the radiation-protective effects of edible mushroom melanin is relatively limited. Future studies should be strengthened to provide more robust theoretical support for its application.

### 4.4. Metal Chelating Ability

Melanin contains anionic groups, providing multiple binding sites for metal ions and conferring the ability to chelate cationic metals. This chelation not only synergistically enhances the biological activity of melanin and meta lion, expanding their application potential, but also effectively reduces the biological toxicity of heavy metal ions through coordination covalent bonding [102,103].

Research has found that melanin from *Auricularia auricula* exhibits strong Fe^2+^ chelating activity [104,105]. In addition, melanin L-25 extracted from *Sporisorium reilianum* exhibits good metal stability, except for Mn^2+^ [74]. The binding of melanin with metal ions is related to pH, melanin type, and the type of metal ion [106,107].

### 4.5. Liver Protection

The liver is a critical metabolic and detoxification organ, and its damage directly affects normal metabolic functions. Research has shown that the occurrence of liver diseases such as hepatitis, liver cancer, cirrhosis, and fatty liver is associated with oxidative stress. Excessive accumulation of reactive oxygen species (ROS) and other byproducts in the liver disrupts the oxidative balance, leading to the peroxidation of biomolecules such as lipids, proteins, and DNA, thereby causing liver damage. CCl_4_, a typical liver toxin, is metabolized by cytochrome P_450_ in the liver, producing large amounts of ROS, trichloromethyl, and their peroxides, which induce oxidative stress-induced liver damage. It serves as an ideal model for studying the antioxidant and liver-protective effects of drugs and is widely used in liver disease research [108,109,110].

Liu et al. [111]. established a mouse model of acute liver injury induced by CCl_4_ to investigate the antioxidant and hepatoprotective effects of *Auricularia heimuer* melanin in vivo. The results showed that compared with the model group, mice in the *Auricularia heimeri* melanin treatment group exhibited significantly reduced serum ALT and AST activity (*p* < 0.01), significantly reduced MDA content in liver tissue (*p* < 0.01), and significantly increased SOD activity (*p* < 0.01). Additionally, the pathological damage in liver tissue was significantly improved. Additionally, melanin from *Auricularia auricula* can protect mice from ethanol-induced liver damage by prolonging the duration of the corrective reflex and shortening the recovery period. Its protective effect may be associated with the activation of nuclear factor E2-related factor 2 (Nrf2) and its downstream antioxidant enzymes [112]. There is also research indicating that the therapeutic effects of *Auricularia auricula* melanin (AAM) may be related to the inhibition of CYP2E1 expression [113]. This suggests that melanin from *Auricularia auricula* may be an effective strategy for alleviating alcohol-induced liver damage. Additionally, the potential effects of AAM on the gut microbiota and liver metabolome of mice exposed to alcohol intake were investigated for the first time, revealing that AAM exhibits potential beneficial effects in alleviating alcohol-induced liver injury, offering promise as a new functional food ingredient [114]. The aforementioned studies all focused on melanin from *Auricularia auricula*, with reports also available for other edible fungi. The protective effects of melanin derived from the fruiting bodies of *I. hispidus* (IHFM) on acute alcoholic liver injury in mice were investigated. The results indicated that IHFM alleviated liver injury in mice by enhancing alcohol metabolism capacity, inhibiting inflammatory responses, and improving antioxidant activity. Additionally, IHFM further exerts its hepatoprotective effects by activating the nuclear factor E2-related factor 2 (Nrf2) signaling pathway and inhibiting the Toll-like receptor 4 (TLR4)/nuclear factor κβ (NF-κβ) signaling pathway [115].

## 5. Applications of Melanins

Compared to melanin from other sources, melanin from edible fungi has significant advantages. Its production is not restricted by seasonal changes, it is inexpensive, easy to maintain, simple to operate, and the reaction conditions are mild. More importantly, the characteristics of edible fungi melanin (such as yield) can be flexibly regulated by adjusting the culture medium components and fermentation conditions. Melanin has a wide range of applications in the food, pharmaceutical, chemical, feed, and agricultural industries, and holds great potential for future development [116,117,118,119]. However, research on the application of melanin derived from edible fungi is currently limited.

Domestic scholars have reported for the first time the use of *Phlebopus portentosus* mycelium fermentation to prepare melanin and analyse its physicochemical properties and have verified the effectiveness of this melanin as a natural hair dye raw material. This provides a theoretical basis and technical reference for the subsequent development of safe and efficient natural hair dyes [17]. Microbial fermentation technology has been widely applied in agricultural product processing and has been proven to be an effective method for improving soybean quality and reducing its antinutritional factors [120,121]. Fermenting soybeans using *Auricularia auricula* significantly reduces the content of antinutritional factors, improves the composition of nutritional components, and enhances the content of polysaccharides, total phenols, total flavonoids, and melanin. The research findings indicate that black fungus holds potential for use in soybean fermentation, capable of enhancing the nutritional quality and bioactivity of the final product. The fermented soybean products obtained through this process possess potential for development as health foods or food additives [122].

Melanin exhibits excellent light and heat stability, compatibility with additives, and biological functions such as radiation resistance, antioxidant properties, and liver protection, making it highly promising and valuable for various applications. Although the synthetic melanin analogue polydopamine holds significant potential in fields such as energy and catalysis, natural melanin derived from edible fungi demonstrates broader application prospects. Owing to its exceptional biocompatibility and biodegradability, it emerges as an ecologically superior alternative in sectors demanding high biosafety standards, including biomedicine and environmentally friendly materials [123,124,125,126,127].

The commercial application of melanin derived from edible fungi hinges not only on its functional advantages but critically on its established safety profile. Given potential risks such as heavy metal accumulation, allergenicity, or unknown metabolites, systematic toxicological evaluations must be conducted at cellular and animal levels for specific strains and products prior to their incorporation into food, pharmaceutical, and cosmetic applications. This establishes the scientific foundation for their practical utilisation [5,25,26,27,28,29,30]. Given the broad application prospects of melanin and the high safety of melanin derived from edible fungi, the use of liquid fermentation technology for production offers significant advantages such as low cost, short production cycles, and ease of industrialization. Whilst the production of edible fungal melanin has demonstrated sound technical feasibility, its transition from laboratory to industrial scale faces multiple challenges, with commercial success fundamentally hinging upon economic competitiveness. Firstly, technical economics present a core challenge: the fermentation process must strike a balance between high cell density, high yield, and controllable energy costs; In downstream extraction, alkaline extraction methods, while low in equipment costs, impose significant environmental treatment burdens. Conversely, enzymatic hydrolysis, despite higher reagent costs, offers long-term advantages in high-end applications due to its mild conditions and superior product quality. Secondly, economies of scale are paramount, necessitating comprehensive assessment of fixed asset investment and operational costs at tonnage-scale production capacity. The product’s natural and safe characteristics must also inform projections of its value potential in premium markets. Moreover, regulatory access presents another key barrier. As a novel raw material, its approval process is more complex and time-consuming than traditional colourants, constituting a significant uncertainty for commercialisation. A simplified financial model can provide preliminary investment return estimates. Overall, despite higher initial investment, edible fungal melanin holds substantial commercial potential if it can precisely target high-value-added markets and continuously optimise the entire process. However, related research is relatively limited, and future efforts should focus on strengthening research in this area.

## 6. Conclusions

Natural melanin possesses multiple unique properties, such as radiation resistance, free radical and reactive oxygen species scavenging, and the ability to chelate various metal ions. As a result, melanin has increasingly become a key component in the development of functional materials, demonstrating broad application prospects and significant development potential across industries such as food, pharmaceuticals, chemicals, feed, and agriculture. Research indicates that by optimizing cultivation parameters and combining them with recombinant technology, melanin production can be effectively enhanced to meet large-scale production demands. However, despite extensive research, its low solubility has hindered the development of efficient extraction processes, while its heterogeneous and complex structure has impeded precise structural analysis, thereby limiting the full exploitation of its biotechnological potential. Notably, existing research has revealed differences in carboxylic acid group content between synthetic and natural melanin, a key factor that may significantly impact their final application efficacy. Therefore, in-depth studies on the biosynthetic mechanisms of melanin and efficient extraction technologies hold both theoretical and practical significance. Among these, melanin derived from edible fungi, due to its sustainability and ease of acquisition, is considered an ideal raw material for industrial production. This review focuses on edible fungus melanin, systematically outlining its extraction methods, characterization techniques, biological activity, and application potential. By comparing and evaluating different analytical methods for melanin types and discussing structural analysis and application prospects, the review aims to help researchers across various fields understand the importance of edible fungus melanin and utilize the information to plan related studies. However, it should be noted that numerous challenges remain to be addressed across the various aspects covered in this review.

## Data Availability

No new data were created or analyzed in this study.

## References

[1] Zhang Y.R., Wang D.W., Chen Y.T., Liu T.T., Zhang S.S., Fan H.X., Liu H.C., Li Y. (2021). Healthy function and high valued utilization of edible fungi. Food Sci. Hum. Well..

[2] Sun Y.N., Zhang M., Fang Z.X. (2020). Efficient physical extraction of active constituents from edible fungi and their potential bioactivities: A review. Trends Food Sci. Technol..

[3] Hamza A., Mylarapu A., Krishna K.V., Kumar D.S. (2024). An insight into the nutritional and medicinal value of edible mushrooms: A natural treasury for human health. J. Biotechnol..

[4] Gosset G. (2017). Biotechnological production of melanins with microorganisms. Bio-Pigmentation and Biotechnological Implementations.

[5] Singh S., Nimse S.B., Mathew D.E., Dhimmar A., Sahastrabudhe H., Gajjar A., Ghadge V.A., Kumar P., Shinde P.B. (2021). Microbial melanin: Recent advances in biosynthesis, extraction, characterization, and applications. Biotechnol. Adv..

[6] Solano F. (2014). Melanins: Skin pigments and much more—Types, structural models, biological functions, and formation routes. New J. Sci..

[7] Eisenman H.C., Casadevall A. (2012). Synthesis and assembly of fungal melanin. Appl. Microbiol. Biotechnol..

[8] Riley P.A. (1997). Melanin. Int. J. Biochem. Cell B.

[9] Liu L.L., Xu H.Y., Gao L., Zhao Y., Wang H.B., Shi N., Guo L.X., Liu P.P. (2022). Application of melanin as biological functional material in composite film field. Sci. Eng. Compos. Mater..

[10] Jalmi P., Bodke P., Wahidullah S., Raghukumar S. (2012). The fungus *Gliocephalotrichum simplex* as a source of abundant, extracellular melanin for biotechnological applications. World J. Microbiol. Biotechnol..

[11] Chongkae S., Nosanchuk J.D., Pruksaphon K., Laliam A., Pornsuwan S., Youngchim S. (2019). Production of melanin pigments in saprophytic fungi in vitro and during infection. J. Basic Microb..

[12] Hill H.Z. (1992). The function of melanin or six blind people examine an elephant. Bioessays.

[13] Eom T., Ozlu B., Ivanová L., Lee S., Lee H., Krajcovic J., Shim B.S. (2024). Multifunctional natural and synthetic melanin for bioelectronic applications: A review. Biomacromolecules.

[14] d’Ischia M., Napolitano A., Ball V., Chen C.T., Buehler M.J. (2014). Polydopamine and eumelanin: From structure–property relationships to a unified tailoring strategy. Acc. Chem. Res..

[15] Luo X., Li X.M., Xue F.Z., Cai Q., Zhu X.F., Wu X.P., Fu J.S. (2023). Physicochemical properties and antioxidant and antibacterial activities of melanin from *Tremella fuciformis* bran. Mycosystema.

[16] Li X.M., Yuan Y., Xue F.Z., Wu X.P., Fu J.S. (2023). Optimization of fermentation conditions for extracellular melanin production from *Inonotus hispidus* and its antioxidant activity. J. Nucl. Agric. Sci..

[17] Wang Z.J., Liu S.J., Wang J. (2023). Antioxidant activity stability and application of melanin obtained from *Phlebopus portentosus*. Acta Edulis Fungi.

[18] Qiu Z.H., Wang S., Zhao J.Z., Gui L.X., Wang X.Y., Cai N., Li H.P., Ren S.H., Li T.L., Shu L.L. (2023). Synthesis and structural characteristics analysis of melanin pigments induced by blue light in *Morchella sextelata*. Front. Microbiol..

[19] Liu X., Hou R.L., Wang D.T., Mai M.X., Wu X.P., Zheng M.F., Fu J.S. (2019). Comprehensive utilization of edible mushroom *Auricularia auricula* waste residue—Extraction, physicochemical properties of melanin and its antioxidant activity. Food Sci. Nutr..

[20] Prados-Rosales R., Toriola S., Nakouzi A., Chatterjee S., Stark R., Gerfen G., Tumpowsky P., Dadachova E., Casadevall A. (2015). Structural characterization of melanin pigments from commercial preparations of the edible mushroom *Auricularia auricula*. J. Agric. Food Chem..

[21] Li X.M., Wu W.Y., Zhang F.P., Hu X., Yuan Y., Wu X.P., Fu J.S. (2022). Differences between water-soluble and water-insoluble melanin derived from *Inonotus hispidus* mushroom. Food Chem. X.

[22] Hou R.L., Liu X., Xiang K.K., Chen L.T., Wu X.P., Lin W.X., Zheng M.F., Fu J.S. (2019). Characterization of the physicochemical properties and extraction optimization of natural melanin from *Inonotus hispidus* mushroom. Food Chem..

[23] Sun S.J., Zhang X.J., Sun S.W., Zhang L.Y., Shan S.K., Zhu H. (2016). Production of natural melanin by *Auricularia auricula* and study on its molecular structure. Food Chem..

[24] Pavan M.E., Lopez N.I., Pettinari M.J. (2020). Melanin biosynthesis in bacteria, regulation and production perspectives. Appl. Microbiol. Biotechnol..

[25] Singh D., Deepshikha., Chaturvedi V., Verma P. (2025). The amazing world of biological pigments: A review on microbial melanins. Dyes Pigment..

[26] Qin Y.P., Xia Y.X. (2024). Melanin in fungi: Advances in structure, biosynthesis, regulation, and metabolic engineering. Microb. Cell Fact..

[27] Song W., Yang H.Y., Liu S., Yu H.H., Li D., Li P.C., Xing R.G. (2023). Melanin: Insights into structure, analysis, and biological activities for future development. J. Mater. Chem. B.

[28] Suthar M., Dufossé L., Singh S.K. (2023). The enigmatic world of fungal melanin: A comprehensive review. J. Fungi.

[29] Roy S., Rhim J.W. (2022). New insight into melanin for food packaging and biotechnology applications. Crit. Rev. Food Sci..

[30] Tran-Ly A.N., Reyes C., Schwarze F.W.M.R., Ribera J. (2020). Microbial production of melanin and its various applications. World J. Microbiol. Biotechnol..

[31] Madkhali N., Alqahtani H.R., Al-Terary S., Laref A., Hassib A. (2019). Control of optical absorption and fluorescence spectroscopies of natural melanin at different solution concentrations. Opt. Quant. Electron..

[32] Araújo M., Viveiros R., Correia T.R., Correia I.J., Bonifácio V.D., Casimiro T., Aguiar-Ricardo A. (2014). Natural melanin: A potential pH-responsive drug release device. Int. J. Pharmaceut..

[33] Ghadge V., Kumar P., Maity T.K., Prasad K., Shinde P.B. (2022). Facile alternative sustainable process for the selective extraction of microbial melanin. ACS Sustain. Chem. Eng..

[34] Gil-Avilés M.D.R., Montes-Avila J., Díaz-Camacho S.P., Picos-Salas M.A., López-Angulo G., Reynoso-Soto E.A., Osuna-Martínez L.U., Delgado-Vargas F. (2019). Soluble melanins of the *Randia echinocarpa* fruit–Structural characteristics and toxicity. J. Food Biochem..

[35] Jiang Q., Luo Z.M., Men Y.Z., Yang P., Peng H.B., Guo R.R., Tian Y., Pang Z.Q., Yang W.L. (2017). Red blood cell membrane-camouflaged melanin nanoparticles for enhanced photothermal therapy. Biomaterials.

[36] Singh N., Prasad K. (2019). Multi-tasking hydrated ionic liquids as sustainable media for the processing of waste human hair: A biorefinery approach. Green Chem..

[37] Sajjan S., Kulkarni G., Yaligara V., Kyoung L., Karegoudar T.B. (2010). Purification and physiochemical characterization of melanin pigment from *Klebsiella* sp. GSK. J. Microbiol. Biotechnol..

[38] Rani M.H.S., Sujith S. (2025). Filamentous fungal-mediated melanin nanoparticles for heavy metal detoxification via bioadsorption: A sustainable approach. Biodegradation.

[39] Zhou J.L., Wang H.Y., Tong L.P. (2023). Extraction, modification, and application of natural melanin. Chin. Sci. Bull..

[40] Wang Z.Y., Chang M.C., Xu L.J., Meng J.L., Zuo N.K., Pan X. (2021). Structural characterization, physicochemical properties of melanin from fruiting body, hyphae and spores of *Ganoderma lucidu*. Biotechnol. Bull..

[41] Yin C.M., Yao F., Wu W., Fan X.Z., Chen Z., Ma K., Shi D.F., Gao H. (2022). Physicochemical properties and antioxidant activity of natural melanin extracted from the wild wood ear mushroom, *Auricularia auricula* (Agaricomycetes). Int. J. Med. Mushrooms.

[42] Medihi N.I., Haiyee Z.A., Sukor R., Raseetha S. (2024). Exploring the functional properties and nutritional values of colored oyster mushrooms species (*Pleurotus*, Agaricomycetes): A review. Int. J. Med. Mushrooms.

[43] Chen B.W., Zhang X.L., Sun X.R., Luo Y., Du H.X., Meng J.L., Xu L.J., Chang M.C. (2017). Extraction, characterisation, stability and antioxidant capacity of *Agrocybe aegerita* melanin. Acta Edulis Fungi.

[44] Liu L.N., Wang R.A., Zhang P. (2015). Extraction and identification of melanin from *Flammulina velutipes*. Acta Edulis Fungi.

[45] Zhang F.P., Xue F.Z., Xu H., Yuan Y., Wu X.P., Zhang J.L., Fu J.S. (2021). Optimization of solid-state fermentation extraction of *Inonotus hispidus* fruiting body melanin. Foods.

[46] Ribera J., Panzarasa G., Stobbe A., Osypova A., Rupper P., Klose D., Schwarze F.W. (2018). Scalable biosynthesis of melanin by the basidiomycete *Armillaria cepistipes*. J. Agric. Food Chem..

[47] Liu Q.M., Xiao J.J., Liu B.T., Zhuang Y.L., Sun L.P. (2018). Study on the preparation and chemical structure characterization of melanin from *Boletus griseus*. Int. J. Mol. Sci..

[48] Wang J.Y., Ma Z.H., Wang C.T., Chen W. (2024). Melanin in *Auricularia auricula*: Biosynthesis, production, physicochemical characterization, biological functions, and applications. Food Sci. Biotechnol..

[49] Chuyen H.V., Nguyen M.H., Roach P.D., Golding J.B., Parks S.E. (2017). Microwave-assisted extraction and ultrasound-assisted extraction for recovering carotenoids from Gac peel and their effects on antioxidant capacity of the extracts. Food Sci. Nutr..

[50] Yuan Y., Yan Y.H., Wu F.Q., Zhang F.P., Huang H.C., Wu X.P., Fu J.S. (2022). Extraction optimization of melanin from *Auricularia heimuer* with complex enzyme and its antioxidant activity analysis. J. Northwest A F Univ. (Nat. Sci. Ed.).

[51] Zou Y., Xie C.Y., Fan G.J., Gu Z.X., Han Y.B. (2010). Optimization of ultrasound-assisted extraction of melanin from *Auricularia auricula* fruit bodies. Innov. Food Sci. Emerg. Technol..

[52] Lu Y., Ye M., Song S., Li L., Shaikh F., Li J.H. (2014). Isolation, purification, and anti-aging activity of melanin from *Lachnum singerianum*. Appl. Biochem. Biotechnol..

[53] Shi Q.W., Yang Z.N., Fan R.H., Chu J.L., Fang C.L., Zhang Y.S., Shi W.T., Zhang Y.J. (2023). Isolation, characterization, and antioxidant activity of melanin from *Auricularia auricula* (Agaricomycetes). Int. J. Med. Mushrooms.

[54] Almeida E.R.V., Melo A.S., Lima A.S., Lemos V.A., Oliveira G.S., Clethen C.F., Bezerra M.A. (2025). A review of the use of central composite design in the optimization of procedures aiming at food chemical analysis. Food Chem..

[55] Hemavathi M., Shekhar S., Varghese E., Jaggi S., Sinha B., Mandal N.K. (2022). Theoretical developments in response surface designs: An informative review and further thoughts. Commun. Stat.-Theory Methods.

[56] Yolmeh M., Jafari S.M. (2017). Applications of response surface methodology in the food industry processes. Food Bioprocess Technol..

[57] Zhang M., Xiao G.N., Thring R.W., Chen W., Zhou H.B., Yang H.L. (2015). Production and characterization of melanin by submerged culture of culinary and medicinal fungi *Auricularia auricula*. Appl. Biochem. Biotechnol..

[58] Ma Y.P., Bao Y.H., Kong X., Tian J.J., Han B., Zhang J.C., Chen X.J., Zhang P.Q., Wang H., Dai X.D. (2018). Optimization of melanin extraction from the wood ear medicinal mushroom, *Auricularia auricula-judae* (Agaricomycetes), by response surface methodology and its antioxidant activities in vitro. Int. J. Med. Mushrooms.

[59] Michael H.S.R., Subiramanian S.R., Thyagarajan D., Mohammed N.B., Saravanakumar V.K., Govindaraj M., Maheswari K.M., Karthikeyan N., Kumar C.R. (2023). Melanin biopolymers from microbial world with future perspectives—A review. Arch. Microbiol..

[60] Lee S.Y., Coutinho J.A., Weingarten M. (2025). Sustainable recovery of microbial-derived natural pigments using deep eutectic solvents: Advances, potential, and challenges. Sep. Purif. Technol..

[61] Das A.K., Sequeira R.A., Maity T.K., Prasad K. (2021). Bio-ionic liquid promoted selective coagulation of *κ*-carrageenan from *Kappaphycus alvarezii* extract. Food Hydrocoll..

[62] Sharma M., Chaudhary J.P., Mondal D., Meena R., Prasad K. (2015). A green and sustainable approach to utilize bio-ionic liquids for the selective precipitation of high purity agarose from an agarophyte extract. Green Chem..

[63] Zhong C., Wang C.M., Huang F., Jia H.H., Wei P. (2013). Wheat straw cellulose dissolution and isolation by *tetra-n*-butylammonium hydroxide. Carbohydr. Polym..

[64] Pombeiro-Sponchiado S.R., Sousa G.S., Andrade J.C., Lisboa H.F., Gonçalves R.C. (2017). Production of melanin pigment by fungi and its biotechnological applications. Melanin.

[65] d’Ischia M., Wakamatsu K., Napolitano A., Briganti S., Garcia-Borron J.C., Kovacs D., Meredith P., Pezzella A., Picardo M., Sarna T. (2013). Melanins and melanogenesis: Methods, standards, protocols. Pigment. Cell Melanoma Res..

[66] Gao Q., Garcia-Pichel F. (2011). Microbial ultraviolet sunscreens. Nat. Rev. Microbiol..

[67] Pralea I.-E., Moldovan R.-C., Petrache A.-M., Ilieș M., Hegheș S.-C., Ielciu I., Nicoară R., Moldovan M., Ene M., Radu M. (2019). From extraction to advanced analytical methods: The challenges of melanin analysis. Int. J. Mol. Sci..

[68] Gessler N.N., Egorova A.S., Belozerskaya T.A. (2014). Melanin pigments of fungi under extreme environmental conditions. Appl. Biochem. Microbiol..

[69] Li C.F., Ji C.M., Tang B.P. (2018). Purification, characterisation and biological activity of melanin from *Streptomyces* sp.. FEMS Microbiol. Lett..

[70] Manivasagan P., Venkatesan J., Senthilkumar K., Sivakumar K., Kim S.K. (2013). Isolation and characterization of biologically active melanin from *Actinoalloteichus* sp. MA-32. Int. J. Biol. Macromol..

[71] Saini A.S., Melo J.S. (2015). One-pot green synthesis of eumelanin: Process optimization and its characterization. RSC Adv..

[72] Tong C.Q., Luo J., Xie C.L., Wei J.H., Pan G.Q., Zhou Z.Y., Li C.F. (2023). Characterization and biological activities of melanin from the medicinal fungi *Ophiocordyceps sinensis*. Int. J. Mol. Sci..

[73] Suárez-Vergel G., Figueroa-Martinez F., Garza-López P.M., García-Ortiz N., Loera O. (2022). DOPA-melanin, component and tolerance factor to heat and UV-B radiation in the conidia of two species of *Cordyceps*. Biocontrol Sci. Technol..

[74] Fu X., Xie M.X., Lu M., Shi L., Shi T.Y., Yu M. (2022). Characterization of the physicochemical properties, antioxidant activity, and antiproliferative activity of natural melanin from *S. reiliana*. Sci. Rep..

[75] De Souza R.A., Kamat N.M., Nadkarni V.S. (2018). Purification and characterisation of a sulphur rich melanin from edible mushroom *Termitomyces albuminosus* Heim. Mycology.

[76] Bonner T.G., Duncan A. (1962). Infra-red spectra of some melanins. Nature.

[77] Ma Y.P., Zhang P.Q., Dai X.D., Yao X.G., Zhou S.Y., Ma Q.F., Liu J.N., Tian S., Zhu J.N., Zhang J.C. (2023). Extraction, physicochemical properties, and antioxidant activity of natural melanin from *Auricularia heimuer* fermentation. Front. Nutr..

[78] Dzierżęga-Lęcznar A., Chodurek E., Stępień K., Wilczok T. (2002). Pyrolysis-gas chromatography-mass spectrometry of synthetic neuromelanins. J. Anal. Appl. Pyrol..

[79] Dzierżęga-Lęcznar A., Kurkiewicz S., Stępień K. (2012). Detection and quantitation of a pheomelanin component in melanin pigments using pyrolysis–gas chromatography/tandem mass spectrometry system with multiple reaction monitoring mode. J. Mass Spectrom..

[80] Greco G., Wakamatsu K., Panzella L., Ito S., Napolitano A., D’Ischia M. (2009). Isomeric cysteinyldopas provide a (photo) degradable bulk component and a robust structural element in red human hair pheomelanin. Pigment. Cell Melanoma Res..

[81] Li X.M., Xie S.Y., Tao Y.X., Wu X.P., Fu J.S. (2023). Pyrolysis products of melanin from *Auricularia heimuer* new cultivar ‘Nonghei No. 2’ analyzed by Py-GC/MS. Mycosystema.

[82] Xue F.Z., Huang H.C., Wu F.Q., Li X.M., Wu X.P., Fu J.S. (2021). Research status and industrial application of fungal melanin. Biotechnol. Bull..

[83] Ye M., Chen X., Li G.W., Guo G.Y., Yang L. (2011). Structural characteristics of pheomelanin-like pigment from *Lachnum singerianum*. Adv. Mater. Res..

[84] Ye M., Wang Y., Guo G.Y., He Y.L., Lu Y., Ye Y.W., Yang Q.H., Yang P.Z. (2012). Physicochemical characteristics and antioxidant activity of arginine-modified melanin from *Lachnum* YM-346. Food Chem..

[85] Saini A.S., Tripathi A., Melo J.S. (2015). On-column enzymatic synthesis of melanin nanoparticles using cryogenic poly (AAM-*co*-AGE) monolith and its free radical scavenging and electro-catalytic properties. RSC Adv..

[86] Tian S.Y., Garcia-Rivera J., Yan B., Casadevall A., Stark R.E. (2003). Unlocking the molecular structure of fungal melanin using ^13^C biosynthetic labeling and solid-state NMR. Biochemistry.

[87] Zhang Y., Wu X.L., Huang C.Y., Zhang Z.H., Gao W. (2022). Isolation and identification of pigments from oyster mushrooms with black, yellow and pink caps. Food Chem..

[88] Paulin J.V., Batagin-Neto A., Graeff C.F. (2019). Identification of common resonant lines in the EPR spectra of melanins. J. Phys. Chem. B.

[89] Cao W., Zhou X.H., McCallum N.C., Hu Z.Y., Ni Q.Z., Kapoor U., Heil C.M., Cay K.S., Zand T., Mantanona A. (2021). Unraveling the structure and function of melanin through synthesis. J. Am. Chem. Soc..

[90] Selvakumar P., Rajasekar S., Periasamy K., Raaman N. (2008). Isolation and characterization of melanin pigment from *Pleurotus cystidiosus* (telomorph of *Antromycopsis macrocarpa*). World J. Microbiol. Biotechnol..

[91] Li B., Li W., Chen X.H., Jiang M., Dong M.S. (2012). In vitro antibiofilm activity of the melanin from *Auricularia auricula*, an edible jelly mushroom. Ann. Microbiol..

[92] Pan Y.Q., Dai D.H., Chen G.C., Hu W.L. (2024). Antioxidant activities of melanin from *Gomphidius viscidus* and its anti-aging, anti-stress injury effects on *Caenorhabditis elegans*. J. Chin. Inst. Food Sci. Technol..

[93] Zhang L.J. (2013). Study on the antioxidant of the *Auriculaia auricuia* melanin. Food Res. Dev..

[94] Zou Y., Yin D.M., Hu W.Z., Jiang A.L., Chen C., Gu Z.X. (2013). Physicochemical properties and antioxidant activities of *Auricularia auricula* melanin. Sci. Technol. Food Ind..

[95] Zou Y., Yin D.M., Jiang H., Du X.W., Liu C.H., Gu Z.X. (2013). Composition analysis and antioxidant activity of *Auricularia auricula* melanin. Food Sci..

[96] Hou R.L., Yuan Y., Xiang K.K., Wu X.P., Lin W.X., Zheng M.F., Fu J.S. (2019). Cellulase and ultrasonic wave synergistic extraction technology of melanin from *Auricularia heimuer* and analysis of antioxidant activity of the melanin product. Mycosystema.

[97] Menichetti A., Mordini D., Montalti M. (2024). Melanin as a photothermal agent in antimicrobial systems. Int. J. Mol. Sci..

[98] Burmasova M.A., Sysoeva M.A. (2017). Chemical composition and biological activity of the BuOH fraction from chaga melanin. Pharm. Chem. J..

[99] Xue J., Wang Y.F., Qi X.F., Zeng W.M., Zhang Y.L., Lei H. (2024). The physicochemical properties and antioxidant and bacteriostatic activities of *Auricularia auricula* melanin modificated by Arginine. J. Food Meas. Charact..

[100] Gauslaa Y., Solhaug K.A. (2001). Fungal melanins as a sun screen for symbiotic green algae in the lichen *Lobaria pulmonaria*. Oecologia.

[101] Revskaya E., Chu P., Howell R.C., Schweitzer A.D., Bryan R.A., Harris M., Gerfen G., Jiang Z.W., Jandl T., Kim K. (2012). Compton scattering by internal shields based on melanin-containing mushrooms provides protection of gastrointestinal tract from ionizing radiation. Cancer Biother. Radiopharm..

[102] Fogarty R.V., Tobin J.M. (1996). Fungal melanins and their interactions with metals. Enzyme Microb. Technol..

[103] Ren Y.L., Yang L., Gao L., Wang F., Shi N., Zhao Y.H., Guo L.X., Wang H.B. (2020). Research progress and application of melanin metal chelate. Mater. Rev..

[104] Zou Y., Hu W.Z., Ma K., Tian M.X. (2015). Physicochemical properties and antioxidant activities of melanin and fractions from *Auricularia auricula* fruiting bodies. Food Sci. Biotechnol..

[105] Zou Y., Yang Y., Zeng B., Gu Z.X., Han Y.B. (2013). Comparison of physicochemical properties and antioxidant activities of melanins from fruit-bodies and fermentation broths of *Auricularia auricula*. Int. J. Food Prop..

[106] Costa T.G., Younger R., Poe C., Farmer P.J., Szpoganicz B. (2012). Studies on synthetic and natural melanin and its affinity for Fe (III) ion. Bioinorg. Chem. Appl..

[107] Mattoon E.R., Cordero R.J.B., Casadevall A. (2021). Fungal melanins and applications in healthcare, bioremediation and industry. J. Fungi.

[108] Ramachandran A., Jaeschke H. (2018). Oxidative stress and acute hepatic injury. Curr. Opin. Toxicol..

[109] Chen X.G., Xu C.S. (2014). Proteomic analysis of the regenerating liver following 2/3 partial hepatectomy in rats. Biol. Res..

[110] Fareed M.M., Khalid H., Khalid S., Shityakov S. (2024). Deciphering molecular mechanisms of carbon tetrachloride-induced hepatotoxicity: A brief systematic review. Curr. Mol. Med..

[111] Liu C.Y., Qi Y., Lv H.Y., Lin W.X., Wu X.P., Fu J.S. (2018). The improvement of *Auricularia heimuer* melanin on acute liver injuried mice. Mycosystema.

[112] Hou R.L., Liu X., Yan J.J., Xiang K.K., Wu X.P., Lin W.X., Chen G.S., Zheng M.F., Fu J.S. (2019). Characterization of natural melanin from *Auricularia auricula* and its hepatoprotective effect on acute alcohol liver injury in mice. Food Funct..

[113] Hou R.L., Liu X., Wu X.P., Zheng M.F., Fu J.S. (2021). Therapeutic effect of natural melanin from edible fungus *Auricularia auricula* on alcohol-induced liver damage in vitro and in vivo. Food Sci. Hum. Well..

[114] Lin Y.C., Chen H., Cao Y.J., Zhang Y.H., Li W.F., Guo W.L., Lv X.C., Rao P.F., Ni L., Liu P.H. (2021). *Auricularia auricula* melanin protects against alcoholic liver injury and modulates intestinal microbiota composition in mice exposed to alcohol intake. Foods.

[115] Yuan Y., Wu F.Q., Zhang F.P., Li X.M., Wu X.P., Fu J.S. (2023). Hepatoenteric protective effect of melanin from *Inonotus hispidus* on acute alcoholic liver injury in mice. Mol. Nutr. Food Res..

[116] Tsouko E., Tolia E., Sarris D. (2023). Microbial melanin: Renewable feedstock and emerging applications in food-related systems. Sustainability.

[117] Morita T., Matsuura T., Izawa H., Kishikawa K., Kohri M. (2024). Melanin upcycling: Creation of polymeric materials from melanin decomposition products. ACS Sustain. Chem. Eng..

[118] Muñoz-Torres P., Cárdenas-Ninasivincha S., Aguilar Y. (2024). Exploring the agricultural applications of microbial melanin. Microorganisms.

[119] Vahidzadeh E., Kalra A.P., Shankar K. (2018). Melanin-based electronics: From proton conductors to photovoltaics and beyond. Biosens. Bioelectron..

[120] He X.Q., Rong P.X., Liu H.Y., Gan B.C., Wu D.T., Li H.B., Gan R.Y. (2022). Co-fermentation of edible mushroom by-products with soybeans enhances nutritional values, isoflavone aglycones, and antioxidant capacity of Douchi Koji. Foods.

[121] Zhang W.J., Deng Z.Y., Liu T.Y., Liang J.F., Liu J. (2024). Fermentation with edible mushroom mycelia improves flavor characteristics and techno-functionalities of soybean protein. Food Biosci..

[122] Cai G.L., Yi X.T., Wu Z.C., Zhou H.B., Yang H.L. (2024). Synchronous reducing anti-nutritional factors and enhancing biological activity of soybean by the fermentation of edible fungus *Auricularia auricula*. Food Microbiol..

[123] Karyani T.Z., Homaei A., Vianello F. (2025). Green pigment hybrid of natural melanin and cellulose nanofibers for sustainable UV-shielding and antioxidant activity. Dyes Pigment..

[124] Pan J.J., Xia Z.P., Deng N.P., Chen L., Zhang H.B., Lu Y., Liu Y., Gao H. (2023). Eumelanin-inspired nanomaterials in electrochemical energy storage devices: A review. Chem. Eng. J..

[125] Al-Shamery N., Heppner F., Dosche C., Morgenschweis S., Bredow T., Wittstock G., Lee P.S. (2025). Functionalized melanin for enhanced energy storage in aqueous and ionic liquid electrolytes. Commun. Chem..

[126] Chen P., He S.B., Wang T.Y., Wang C.C., Tao J.R., Li Y.W. (2025). Melanin-like nanofibers with highly ordered structures achieve ultrahigh specific electromagnetic interference shielding efficiency. Nat. Commun..

[127] Deng R.T., Chen W., Zhu H.J., Li Y.X., Ou Y.L., Wang J., Ruan Q., Zhang X.Y., Zhang J.B., Zhang Y.X. (2025). Molecular engineering of melanin for enhanced biological γ-ray protection. Nat. Commun..

